# Participación de enfermería en la vigilancia y prevención de la resistencia antimicrobiana

**DOI:** 10.15649/cuidarte.2980

**Published:** 2023-03-29

**Authors:** Antonio Pazin-Filho

**Affiliations:** 1 . Faculdade de Medicina de Ribeirao Preto - Universidade de Sao Paulo, Brasil. Email: apazin@fmrp.usp.br Universidade de São Paulo Universidade de Sao Paulo Brazil apazin@fmrp.usp.br

 La infección nosocomial tiene sus orígenes en el propio nacimiento de los hospitales. Sin embargo, antes del desarrollo de los antibióticos, los hospitales eran instituciones para los pobres necesitados de cuidado, con una elevada tasa de mortalidad, causada no solamente por las enfermedades de base para las cuales no había tratamiento, sino incluso por las condiciones que propiciaban la infección nosocomial^1^.

La Guerra de la Crimea en 1854 fue la primera cubierta por la prensa, que destacaron las malas condiciones en el cuidado de los soldados heridos en combate, con tasas de mortalidad alrededor del 42%. La presión popular inglesa incentivó a que Florence Nightingale (1820-1910) se desplazase hasta Crimea y las medidas sanitarias implementadas derrumbaron la tasa de mortalidad hasta el 2% rápidamente^2^^,^^3^. Mientras aún no se conocía la Teoría de los Gérmenes, es posible afirmar que el éxito ocurrió a causa del control de la infección en las heridas. Esos esfuerzos garantizaron a Florence Nightingale la notoriedad para que le encargasen el perfeccionamiento de las condiciones sanitarias hospitalarias cuando regresó a Inglaterra, fortaleciendo la asepsia (la limpieza seguida por la esterilización de los equipos para procedimientos) y la antisepsia (el uso de substancias esterilizantes). Uno de sus cambios fue justamente el desarrollo de la Enfermería como profesión, que ya nace asociada al control de las infecciones.

La transformación del hospital se completó con el descubrimiento de los antibióticos en la década de 1940, el nacimiento de la Anestesia y los cambios sociales de las ciudades. Nacía el hospital moderno, capacitado para realizar procedimientos quirúrgicos y tratamientos que lo convirtieron en el centro de la Salud actual, hecho que se incrementó después de los reportes de Flexner acerca de las condiciones de enseñanza en las facultades de medicina en los EEUU^4^.

Los antibióticos impactaron de modo tan exitoso las tasas de infección en los principios de su utilización, que el énfasis en las otras medidas de prevención disminuyó^5^. Además, la incidencia de resistencia a los antibióticos fue detectada tempranamente, casi al mismo tiempo que su introducción. En conjunto, esos dos puntos añadidos al incremento de procedimientos invasivos y al uso indiscriminado en otras áreas fuera de la Salud, como la ganadería, contribuyeron para que la resistencia a los antibióticos se convirtiese en la calamidad que vivimos actualmente.

Mientras se sigan buscando nuevos antibióticos, mucho de la investigación es dedicada a la búsqueda de otras soluciones como las vacunas, inmunoterápicos, nanobios, terapia fágica, células madre y moléculas de adhesión. Sin embargo, aunque mucho se haya descubierto, aún no estamos preparados para aplicarlos a la clínica diaria^6^.

Como consecuencia, se incrementan y mejoran la correcta utilización de las técnicas de asepsia y antisepsia, en las cuales la participación de la Enfermería es clave. Las comisiones de infección hospitalaria están identificando y corrigiendo las técnicas de lavado de las manos, la correcta utilización de guantes, mascarillas y otras herramientas de protección individual, las reglas de aislamiento respiratorio y de contacto. Centrarse en el proceso de utilización de esas técnicas es el punto común a ser trabajado por la enseñanza continuada^7^.

Además, la Enfermería está involucrada en el cambio fundamental de una actitud pasiva, basada en la documentación de indicadores de infección, a una actitud activa en que se busca la prevención. Programas de Optimización del Uso de los Antimicrobianos (“Antibiotic Stewardship”) incluyen herramientas de control como la Dosis Diaria Definida (DDD) para el control del consumo de los antibióticos, la identificación de campeones (“champions”) en el acompañamiento de los procesos diarios, el cambio de los procedimientos de limpieza, la incorporación de nuevas tecnologías de antisepsia y del planteamiento de reformas en la estructura hospitalaria. Estos programas demandan personal entrenado y es un campo de especialización para todos los profesionales de la Salud. Aunque esos cambios ya sean preconizados por la OMS y empleados en países desarrollados, en los países en desarrollo aún son incipientes^8^^,^^9^.

Sin embargo, el punto clave es no cometer el mismo error que en el pasado, cuando nos descuidamos de las medidas de asepsia y antisepsia a causa del éxito de los antimicrobianos. Nuestra mejor estrategia no es una u otra técnica, sino su aplicación en conjunto y de modo coordinado, por personal entrenado y bajo constante vigilancia. Las lecciones de Florence Nightingale continúan verdaderas y necesarias.
